# Positive Effects of the Caregiver Skill Training Program on Children With Developmental Disabilities: Experiences From Serbia

**DOI:** 10.3389/fpsyt.2022.913142

**Published:** 2022-06-03

**Authors:** Nenad Glumbic, Roberto Grujicic, Sanja Stupar, Suncica Petrovic, Milica Pejovic-Milovancevic

**Affiliations:** ^1^Faculty of Special Education and Rehabilitation, University of Belgrade, Belgrade, Serbia; ^2^Department for Children and Adolescents, Institute of Mental Health, Belgrade, Serbia; ^3^Serbian Society of Autism, Belgrade, Serbia; ^4^Faculty of Medicine, University of Belgrade, Belgrade, Serbia

**Keywords:** developmental delays, parent-mediated intervention, support, parenting skills program, parent-child relations, public health, Serbia

## Abstract

**Background:**

Intervention programs for children with developmental disabilities increasingly target caregiver training to implement effective strategies for child development. Research conducted in different countries shows that the Caregiver Skills Training Program (CST) developed by the World Health Organization and Autism Speaks could also be a recommended intervention.

**Methods:**

The pre-pilot phase included seven, and the pilot phase included 29 families of children with developmental disabilities trained to implement the intervention program. The caregivers were asked to complete the Autism Treatment Evaluation Checklist at the beginning and at the end of the program.

**Results:**

In the pre-pilot phase, the Wilcoxon signed-rank test determined a statistically significant improvement in Speech, Language and Communication (*z* = −2.99, *p* < 0.05) and Health/Physical/Behavior (*z* = −2.375, *p* < 0.05) after caregiver participation in the training program. In the pilot phase, the paired *t*-test also determined a statistically significant improvement in Speech, Language and Communication between the first (M = 24.52, SD = 5.57) and the second testing (M = 25.66, SD = 6.11), t_(28)_ = −2.29, *p* < 0.05, as well as a significant improvement between the first (M = 36.62; SD = 7.15) and the second testing (M = 35.38; SD = 5.91), t_(28)_ = 2.11, *p* < 0.05 in Health/Physical/Behavior. Eta squared values (0.16 and 0.14) indicate that the intervention effect was significant. No differences were determined in Sociability and Sensory/Cognitive Awareness between the first and the second testing.

**Conclusion:**

The initial results of the Caregiver Skills Training Program are encouraging. For this program to be recommended as an evidence-based intervention, further research should be conducted on larger samples, controlling possible intervening variables.

## Introduction

Children with developmental delays (DD) need continuous lifelong support, from birth to adulthood. In many countries, especially in low and middle-income countries (LMIC), adequate services are poorly accessible or completely inaccessible to this vulnerable group (1). The children with DD from LMIC usually deprived of early recognition and community-based interventions, but sometimes also inclusive and adequate education, health care, evidence-based interventions, and rehabilitation (2). Moreover, the support of caregivers and the whole family is equally important, especially for families with restricted access to such services (3). Interventions implemented in all resources must be evidence-based and tailored for children with DD. Out of all implemented strategies and methods for children with DD, early recognition and timely, evidence-based, individual-oriented interventions have performed best (2, 4). In order to provide a timely diagnosis and refer children to a certain intervention, all service providers for children and families (e.g., healthcare providers, social workers, teachers and employees in the educational system), need to be educated and trained.

However, sometimes even with appropriate support systems and care providers, there is not a sufficient improvement in child's behavior and development. This can happen in cases where experts are mainly focused on delivering interventions to children, neglecting the role of caregivers in overall treatment (5, 6). All service and care providers should recognize the fact that the caregivers of children with DD spend the most time with their child and can thus carry out most of the treatment and support. In contrast, if there is no cooperation with parents, relatively little can be achieved in terms of improving the child's wellbeing and independency (6). Therefore, the caregivers of children with DD should be provided with continuous information and assistance in understanding the diagnosis, as well as be empowered to participate in the decision-making process for intervention design; parents should be given a chance to acquire the skills needed to help in the process of the child's treatment.

Serbia is a country of ~7 million people where about 1 million people are aged 0–17 (7). Like most of the LIMC, Serbia has a significant problem in providing appropriate, evidence-based mental health care for children with DD, mostly due to the lack of staff, research and infrastructure (8). Even if the needed support is available in certain areas, families face additional problems, including restricted access, financial issues, time constraints, etc. (3, 9). In rural areas the problem is most visible. Despite the fact that approximately half of the population resides in rural areas, almost all kinds of professional support for children with DD are inadequate (9).

One of the most recognized parent-based education programs to date is Caregivers' Skills Training (CST) developed by Autism Speaks (AS) and the World Health Organization (WHO) (10). The training is intended for parents and caregivers of children with DD. This program offers the opportunity for caregivers to fully participate in their child's treatment, as well as to acquire skills for supporting their child's development (10). The program consists of nine group sessions and three individual home visits. The training is designed to teach the caregiver how to use every day play and home routines as opportunities for learning and development (11). This program was initiated in Serbia as part of the overall initiative to strengthen the Early Childhood Intervention (ECI) system in the country. Care providers and policy-makers in Serbia recognized the importance of a family-focused approach and the vital role of caregiver education in the field of ECI. Recognizing the potential role of CST, the professionals involved in ECI programs welcomed it enthusiastically.

To date, the CST program has been implemented, or is in the process of implementation, in more than 30 countries all over the world, and many of these countries are LIMC (12). The CST has shown to be a promising intervention in LIMC due to the fact that it doesn't require the involvement of specialists or other high profile experts (it can be even delivered by educated parents or volunteers), it doesn't require high material/financial resources or expensive equipment. The studies conducted in these countries indicate that CST could be the intervention which can be implemented, culturally adapted and sustainable for many LIMC (13–15).

The main aim of this research is to evaluate the effects of the CST program on different domains of child's development. In addition, this research aims to evaluate the process of implementation, cultural appropriateness and parental opinions on the CST program in Serbia.

## Materials and Methods

### Participants

The CST program in families of children with developmental disabilities was conducted in a pre-pilot and a pilot phase. The pre-pilot phase included seven and the pilot phase 29 families. There was one child with developmental disabilities in each family. Tables 1, 2 show demographic data on the caregivers and children with developmental disabilities from both research phases.

**Table 1:** The socio-demographic data of caregivers.

	**Pre-pilot (*n* = 7)**	**Pilot (*n* = 29)**
	***n***	***n* (%)**
**Main caregiver**
Mother	5	27 (93.1)
Father	2	2 (6.9)
Age M (SD)	40.29 (6.95)	35.76 (5.2)
**Education**
Primary	0	1 (3.4)
Secondary	2	13 (44.8)
University	5	15 (51.7)
**Marital status**
Single	0	1 (3.4)
Married (partner)	6	26 (89.8)
Divorced	1	1 (3.4)
Widowed	0	1 (3.4)
**Place of living**
Rural	1	6 (20.7)
Urban	6	23 (79.3)
**Working outside the home**
No	2	11 (37.9)
Part-time	0	4 (13.8)
Full time	5	14 (48.3)
**People who are cared for**
Children M (SD)	2.14 (1.17)	1.62 (0.6)
Adults M (SD)	0	0.14 (0.5)

**Table 2:** The demographic data of children.

	**Pre-pilot (*n* = 7)**	**Pilot (*n* = 29)**
	***n***	***n* (%)**
**Sex**
Male	5	20 (69.0)
Female	2	9 (31.0)
Age M (SD)	5.96 (1.6)	4.11 (1.1)
**Diagnosis**
Intellectual disability	0	1 (3.4)
Communication disorder	1	5 (17.2)
Autism spectrum disorders	4	8 (27.5)
Motor disorder	0	2 (6.9)
Cerebral palsy	0	2 (6.9)
Mixed specific developmental disorder	2	7 (24.1)
Developmental delay	0	1 (3.4)
**Received support**
Health professional (medication)	2	8 (27.6)
Health professional (no medication)	5	9 (31.0)
Special education	6	16 (55.1)
Speech therapy	6	14 (48.2)
Behavioral therapy	0	5 (17.2)
Information on child's problem	2	0 (0.0)
Information on services	0	4 (13.7)

Five boys and two girls, 4–8.5 years of age (M = 5.96; SD = 1.61), participated in the pre-pilot phase, while 20 boys (69%) and nine girls (31%), 2–9 years of age (M = 4.11; SD = 1.92), participated in the pilot phase of the study. Both research phases included a convenience sample of caregivers who requested health service support due to developmental disabilities in children (most frequently autism spectrum disorders - ASD, mixed specific developmental disorder, and communication disorder). At the time of research, three children showed significant developmental delay but had not yet been diagnosed.

### Procedure

The program was divided into two consecutive phases: pre-pilot and pilot phases. In the first (pre-pilot) phase, the master trainers were trained and delivered the training to the parents. ‘Master trainers' is a term determined by the program which describes experts who are in charge of implementation, supervision and delivery of the program in their country. After their extensive training by the founders of CST, the master trainers are able to deliver the CST to other experts in their country (in later text: facilitators) who will continue their work with the families under supervision. Each country has a certain number of trained master trainers who are usually experts in child mental health and development. In our country, eight master trainers (six women and two men), 28–49 years of age (M = 39.25; SD = 8.515), participated in the training program. All master trainers were experts in mental health or health associate professionals in primary healthcare. Two of them were employed in private healthcare institutions, and six worked in public healthcare institutions. Given the differences in their age, the master trainers were expected to have different work experience ranging from four to 22 years. Also, they spent between two and 22 years working with children. Two master trainers had no previous experience in training parents, while six did.

In the second (pilot) phase, each master trainer trained two facilitators to conduct the program with the families. After their training, the facilitators delivered the program to the families under the supervision of a master trainer. Apart from one facilitator who worked in a public education institution, all others were employed in a public healthcare institution.

In each phase, the program was comprised of nine parental group sessions with three home sessions.

### Instruments

All instruments and measures created for CST and used in this research were recommended and provided by the WHO and AS. The measures were translated, back-translated and adapted to Serbian language.

#### Background Measures

Information on master trainers, caregivers, and facilitators, partially presented in the Participants and Procedures sections, was collected using CST background measures: Demographic and professional background information for facilitators and Information about caregivers: Demographic and service history information.

#### CST Program Implementation Measures

The Post session Feedback Form—Caregivers and the Home Visit Participant Feedback Form were used to assess the implementation of the CST program. After each session, the caregivers completed the Post session Feedback Form stating their observations and impressions. The Form included 14 questions requiring the caregivers to assess the degree of understanding, the relevance of the information received, the usefulness of key messages and tips, the usefulness of advice in achieving their own program goals, learning activities, duration of the session and its parts, etc.

In addition to expressing their views about the sessions, caregivers were asked to share their experiences after each home visit with master trainers/facilitators by completing the Home Visit Participant Feedback Form. This Form included only five questions, requiring caregivers to assess the duration and benefits of the home visit, and the benefits of the feedback received during the recording. Open-ended questions were used to collect additional information about whether video-recording was acceptable from the caregivers' perspective and their suggestions for improving home visits.

#### Outcome Measures

The Autism Treatment Evaluation Checklist (ATEC) (16) was used at the beginning and the end of the program to determine the effects of the CST program on children with developmental disabilities. ATEC consists of four subtests. The first subtest, Speech/Language/Communication, includes 14 items. The given answers are 1—not true, 2—somewhat true, or 3—very true, and a higher score indicates better-developed communication skills. The Sociability subtest includes 20 items with three possible answers: 1—not descriptive, 2—somewhat descriptive, and 3—very descriptive. The same answers are provided for the Sensory/Cognitive Awareness subtest that includes 18 items. The difference is that a higher score indicates greater difficulties on the Sociability subtest, while it indicates better-developed skills on the Sensory/Cognitive Awareness subtest. The last subtest, Health/Physical/Behavior, includes 25 items graded on a four-point Likert scale (1—not a problem, 2—minor problem, 3—moderate problem, 4—serious problem).

#### Statistical Analysis

The statistical analysis was performed in IBM SPSS Statistics version 27. Due to the small sample in the pre-pilot research phase, the difference in the participants' achievements on ATEC between the first and the second testing was determined by the Wilcoxon signed-rank test. The paired samples *t*-test was used to compare the first and the second test results in the pilot phase. Repeated measures two-way ANOVA was used to determine whether the participants' scores on ATEC in the pilot phase differed depending on whether the program was implemented in person or online. The repeated factor tested the difference between the two measurements, and the factor that was not repeated referred to the difference in the way the training was implemented.

#### Ethics

This research has been approved by the Ethics Committee of the Institute of Mental Health in Serbia. All participants have signed the Informed Consent Form prior to inclusion in the research.

## Results

The main aim our research was to evaluate the effects of the CST on child's development and to evaluate the appropriateness of the program in our country. To achieve this, we collected data from caregivers in two subsequent phases on their attitudes toward the program as well as their views on how the program effected their child's development. In this section we will present the obtained quantitative and some qualitative data.

### Parental Feedback

The data obtained using the Post session Feedback Form—Caregivers, after each session, and Home Visit Participant Feedback Form, after each home visit, could only be processed descriptively. All scores were very high. Thus, due to the small variability of the obtained data, the satisfaction with individual sessions and home visits was not observed as a control variable in further analysis of the training outcomes. The degree of understanding, the relevance of the information received, the usefulness of key messages and tips, and the usefulness of advice in achieving program goals were assessed by a five-point Likert scale in the Post session Feedback Form. The average scores in both research phases ranged between 4.29 and 5.00. Participants listed different key messages and tips they considered the most or the least significant in each session, but the general tendency was to choose the first ones in a line. With regard to learning activities, group discussion and demonstration were most often mentioned as the favorite ones. More than 80% of the participants positively evaluated the duration of individual sessions and activities. Over 90% of the caregivers felt very or somewhat willing to apply the advice received during sessions at home. Depending on the session, between 80 and 95% of the participants stated that the messages they received during sessions did not contradict what they or their family members considered good or significant.

In addition to expressing their views on the sessions, the caregivers were asked to share their experiences with master trainers/facilitators after every home visit. Almost all participants stated that the home visit lasted as long as necessary, that it was useful, and that the feedback obtained by video recording was valuable. In both research phases, the caregivers were positive about the fact that video recording was an integral part of the program.

### Effects on the Child's Development

The Tables 3, 4 show the scores on four domains of ATEC scale measured before and after the program. The four domains are as follows: Speech/Language/Communication, Sociability, Sensory/Cognitive Awareness and Health/Physical/Behavior. The Table 3 shows the results from the families that participated in the pre-pilot phase (*N* = 7), while the Table 4 shows the results from the families in the pilot phase (*N* = 29).

**Table 3:** Scores of children with developmental disabilities on ATEC at the beginning and the end of the CST pre-pilot program.

**ATEC subscales**	**Baseline (*n* = 7)**		**After intervention (*n* = 7)**		***Z***	***p***
	**M**	**SD**	**M**	**SD**		
Speech/language/communication	29.14	6.41	31.57	6.00	−2.232	**0.026**
Sociability	30.86	3.18	29.14	5.49	−1.382	0.167
Sensory/cognitive awareness	40.86	4.49	43.43	6.27	−1.572	0.116
Health/physical/behavior	40.29	14.47	36.86	13.80	−2.375	**0.018**

*The bolded values indicate the values to be statistically significant (p <0.05)*.

**Table 4:** Scores of children with developmental disabilities on ATEC at the beginning and the end of the CST pilot program.

**ATEC subscales**	**Baseline (*n* = 29)**		**After intervention (*n* = 29)**		***t***	**df**	***p***
	**M**	**SD**	**M**	**SD**			
Speech/language/communication	24.52	5.57	25.66	6.11	−2.296	28	**0.029**
Sociability	30.31	7.19	29.14	6.41	1.177	28	0.249
Sensory/cognitive awareness	41.59	7.66	41.72	7.92	−181	28	0.857
Health/physical/behavior	36.62	7.15	35.58	5.91	2.109	28	**0.044**

*The bolded values indicate the values to be statistically significant (p <0.05)*.

The Wilcoxon signed-rank test determined significant improvements in Speech/Language/Communication (*Z* = −2,232, *p* < 0.05) and Health/Physical/Behavior (*Z* = −2,375, *p* < 0.05), with a large effect size by Cohen's criteria (*r* = 0.60 for Speech/Language/Communication and *r* = 0.63 for Health/Physical/Behavior).

Similar results were obtained in the pilot phase on a somewhat larger sample. Significant improvements were found in Speech/Language/Communication (*t* = −2.296, *p* < 0.05, Eta squared = 0.16) and Health/Physical/Behavior (*t* = 2.109, *p* < 0.05, Eta squared = 0.14), while there were no significant differences between the first and the second testing on the remaining two subscales. Eta squared values indicate that the intervention effect was significant.

Two-way ANOVA did not indicate the interaction between the two factors (repeated assessment and the way the program was implemented), both in Speech/Language/Communication (*F* = 0.900, *p* = 0.351, Eta squared = 0.032), and in Health/Physical/Behavior (*F* = 0.854, *p* = 0.364, Eta squared = 0.031). This suggests that the effects of the program did not depend on whether the training was conducted in person or online.

## Discussion

This research has been conducted as part of the implementing process of the CST project in Serbia. This process was composed of two consecutive phases including the pre-pilot and pilot phases. In the first part of the pre-pilot phase, eight master trainers (MTs) were trained by the representative of AS. All MTs are experts in the field of mental health or health associates in primary health care. The training was supervised by the representatives of AS, who had many years of experience in the CST program implementation. The training of master trainers (TOMT) lasted for 5 days. It was conducted in person and included explaining procedures, key recommendations and advice, role-playing, modeling expected behavior, and direct work with patients. The other part of the TOMT was designed to deliver the whole CST program to the parents. In other words, the trained MTs delivered the whole program to the families of children with DD. Their activities were recorded, thus allowing the supervision of experts from AS.

The CST training of parents was provided in nine parental group sessions and three home visits. The entire training and research material (facilitator guides, home visit facilitator guides, participants booklets for each session, etc.) was translated into Serbian. Then, a material adaptation strategy was developed in direct meetings with the representatives of AS. The Adaptation Team and Adaptation Advisory Group were formed to work together on linguistic and cultural material adaptation. The Adaptation Advisory Group included experts in working with children with DD and neurodevelopmental disorders and their families.

In the pilot phase, the certified MTs trained the facilitators to implement training. The final goal was that the facilitators could then work directly with families of children with DD under the supervision of the MTs. The facilitators had different professions: nurses, educators, health associate professionals in primary healthcare, community nurses, mental health experts, etc.

Most of the respondents from both phases of the research were mothers. This was not surprising, as in Serbia childcare responsibilities are traditionally the job of mothers. Contrary to expectations, two participants in each research stage were fathers. The study that pre-piloted the CST in Ethiopia had a similar result with a low number of fathers (13). The literature review findings reveal a significant and positive impact of father involvement on the child's development, and especially the development of cognitive skills (17). For this reason, we should also aim to include more fathers in the program.

The majority of families who participated in the study were married parents. We do assume that married parents are more willing to participate in training and education processes as sharing caring responsibilities allows greater time for participation. However, we also need to highlight the importance of single-parent and divorced families that already have a significant childcare burden, and even more so if they are a single parent to a child with DD. This is especially the case with single mothers, who face additional burdens associated with gender inequalities typical in the region (e.g., financial problems, professional struggles, lack of time, lack of social support, etc.) (18). A United Nations Children's Fund (UNICEF) study from 2018 revealed that patriarchal gender norms in Eastern Europe and the Balkan region heavily influenced parenting, especially affecting single mothers (19). In the future, married families should not be the only focus of the program. We need to find a way to provide a chance for single parent families, and single mothers, to participate fully in the CST program.

This research was conducted in the centers that implemented the CST, all of which are located in urban areas (i.e., four biggest cities in Serbia). The resulting sample hence only included participants from urban surroundings. However, 46% of families in Serbia live in rural surroundings with very limited resources and access to health and social care (7). Because families from urban surroundings already have better access to services and treatments for children, more families of children with DD from rural surroundings need to be included in future caregiver trainings.

The outbreak of the COVID-19 pandemic during the training program affected the implementation of group sessions and home visits. During the pandemic, the CST program was adapted in order to help in switching the training to online setting (12). Some MTs and facilitators decided to continue the training online. Five families underwent in-person training in the pre-pilot phase, while two families participated in online training. However, as the virus spread over time, the need for online training increased. Only eight (27.6%) families had in-person training in the pilot phase, while 21 (72.4%) families used a video-conferencing system. Although the pilot phase was initially planned to last for 12 weeks, it was extended to 1 year due to the pandemic. As the CST program was not initially designed to be delivered online, some major adaptations had to be performed along the way. If it is to be fully digitalised, the program needs to be further adapted. Additionally, it is possible that families from rural surroundings will require support in handling fully digitalised modes of training. These adaptations were targeted at the delivery of practical exercises and group discussions which were not readily conductable in an online setting.

Most of the families included in the sample received some kind support, commonly in the form of special education, speech therapy and medical attention. In our previous research, we have shown that over ninety per cent of families of children with DD reported that additional support from all systems is important and necessary (3). Other studies confirmed our findings, showing that there is still a wide range of barriers that prevent families from accessing needed support, even with the implementation of strategies for increasing the accessibility of services in LIMC (9). We have also previously identified some predictors of bad overall satisfaction with the provided support, and one of the most significant predictors was parent frustration with accessing services. More positive feedback was gathered from parents who received assistance in managing their child's needs and who had access to an expert as a source of information on autism (3). The CST provides a chance for parents to acquire enough information both from experts and other parents in order to increase their confidence and gain new skills. Promising results from this study lead us to believe that this program can offer a long-term solution to the problem of restricted access and parental frustration in our country.

The majority of the participants evaluated the program positively, as very successful and important for them; the degree of understanding, the relevance of the information received, the usefulness of key messages and tips, and the usefulness of advice in achieving program goals were evaluated between 4.29 and 5.00. This feedback can be interpreted as an endorsement of the CST program, as very well-organized and informative and useful for parents. Our results indicate that there is now an extremely practical, step by step program in Serbia which allows parents to feel understood, appreciated, and respected. This program allows parents to feel as the most valuable actor in their child's treatment and not only a passive observer in the process. It provides parents with very high professional support, as well as with much needed emotional support. The research from other countries that implemented the CST reports similar results, especially in the LIMC (11, 13, 14).

Group discussions and practical exercises were most often voted as favorite parts of the training. Previous literature reviews show that group-based parenting programs show positive and promising results in improving emotional and behavioral functioning of young children as well as parental confidence, knowledge and wellbeing (20, 21). A new study conducted specifically on CST in Italy also shows promising results in the same context (11). From our experience, group support is crucial, mainly because parents can feel understood and accepted. This is a good starting point for future work with families that highlights the importance of organizing parental support groups of children with DD. However, it is important to be mindful of the duration of sessions; too short could be insufficient, and too long could be inaccessible for working parents. CST training obtained balanced timing and participants positively evaluated the duration of individual sessions and activities.

Even though the groups were small to be analyzed in more detail, the parents believe that CST helped them in improving Speech/Language/Communication and Health/Physical/Behavior of their children. This is not surprising, as the program is specifically designed to improve the above-mentioned domains of functioning; out of nine sessions, two sessions are dedicated to understanding and improvement of the child's communication, and two sessions for understanding and controlling the child's behavior. Research conducted on the CST program in other countries reports similar results (11, 13, 14). According to our previous research, the primary concern of parents in our country was related to the child's interaction and communication (3). This finding can also partly explain high rates of parental satisfaction in our county, as the program aims to target the associated concerns with interaction and communication.

### Limitations

This research had several limitations. The first is a limited sample size. This was partly due to fact that this was an implementation (pilot) phase of the program which had a limited number of participants. In addition, due to the COVID pandemic outburst, the research suffered a certain dropout of families before the ending of the program. Namely, some families had technical difficulties, some had health-related issues, and some did not want to continue the program due to the shift of priorities in the time of crisis. All of this also resulted in a limited amount of collected data delivered to the research team.

The adaptation of the program in Serbia took place mainly in urban areas, in clinical settings and delivered mainly by professionals with long years of experience in working with families of children with DD. We are aware that delivering the program in rural settings by non-specialists would possibly have different benefits and recommendations, but also possible additional challenges that were not discovered in this round of research.

## Conclusion

Caregiver's education is a process of systematic provision of necessary information to caregivers which can provide them with the specific knowledge and skills for encouraging their child's development and competence. In the past decade, some systematic reviews, mostly from high-income countries, suggest that caregivers are able to learn and acquire the skills needed to deliver intervention strategies to their children with DD (22, 23). Education and mastering specific skills can significantly improve the mental health of caregivers, the caregiver-child relationship and the process of adapting to new conditions (5, 24). Reviews show that families can significantly benefit from these types of interventions even if they are short and of low intensity (2, 25, 26).

With the introduction of the CST program in Serbia, local partners build the capacities of relevant institutions and professionals. This type of knowledge and skills will be applicable in working with all families of children with disabilities, so that professionals educated through this program remain a permanent resource for participating institutions. The pilot project is implemented in three cities in Serbia (Belgrade, Novi Sad, Nis), in four different health care institutions (primary and tertiary) dealing with the DD of children and their mental health. Steps have been taken to train facilitators from other cities in Serbia, making the program available to more families. With the support of partners from the respective governmental institutions, the program will become a regular service in institutions dealing with early intervention and developmental delay in children, and to will be cost free for the families.

Findings from the application of CST in Serbia indicate that the program could potentially aid a wider variety of parents and caregivers, and thus that the form of its application (thorugh group discussions, online, etc.) can encompass a greater number of parents, providing them adequate support for working with children and as well as for accepting their child's DD. Our indicative results provide the argument for its wider implementation, but it requires consistent advocacy efforts in order to secure the support of decision-makers. Only in that way the CST in Serbia can be widely implemented and sustained over time.

## Data Availability Statement

The raw data supporting the conclusions of this article will be made available by the authors, without undue reservation.

## Ethics Statement

The studies involving human participants were reviewed and approved by Ethics Committee of the Institute of Mental Health. The patients/participants provided their written informed consent to participate in this study.

## Author Contributions

NG contributed with technical support, data collection, literature research, writing and reviewing of the article, and statistical analysis. SP, RG, and SS contributed with data collection, literature research, result analysis, interpretation, writing, and reviewing process. MP-M contributed in the writing and reviewing process, the interpretation of the results, and expertise. All authors contributed to the article and approved the submitted version.

## Funding

The implementation of the CST Program and this research was supported by UNICEF Serbia and HELP—Mission to the Republic of Serbia. All authors received funding from these two organizations.

## Conflict of Interest

The authors declare that the research was conducted in the absence of any commercial or financial relationships that could be construed as a potential conflict of interest.

## Publisher's Note

All claims expressed in this article are solely those of the authors and do not necessarily represent those of their affiliated organizations, or those of the publisher, the editors and the reviewers. Any product that may be evaluated in this article, or claim that may be made by its manufacturer, is not guaranteed or endorsed by the publisher.
